# Development and virucidal activity of a novel alcohol-based hand disinfectant supplemented with urea and citric acid

**DOI:** 10.1186/s12879-016-1410-9

**Published:** 2016-02-11

**Authors:** Georgios Ionidis, Judith Hübscher, Thomas Jack, Britta Becker, Birte Bischoff, Daniel Todt, Veronika Hodasa, Florian H. H. Brill, Eike Steinmann, Jochen Steinmann

**Affiliations:** Oro Clean Chemie AG, Allmendstrasse 21, 8320 Fehraltorf, Switzerland; Dr. Brill + Partner GmbH, Institute for Hygiene and Microbiology, Norderoog 2, 28259, Bremen, Germany; Institute for Experimental Virology, TWINCORE Centre for Experimental and Clinical Infection Research; a joint venture between the Medical School Hannover (MHH) and the Helmholtz Centre for Infection Research (HZI), Feodor-Lynen-Str. 7, 30625 Hannover, Germany

**Keywords:** Hand disinfectant, Virucidal activity, Alcohol, Viruses

## Abstract

**Background:**

Hand disinfectants are important for the prevention of virus transmission in the health care system and environment. The development of broad antiviral spectrum hand disinfectants with activity against enveloped and non-enveloped viruses is limited due to a small number of permissible active ingredients able to inactivate viruses.

**Methods:**

A new hand disinfectant was developed based upon 69.39 % w/w ethanol and 3.69 % w/w 2-propanol. Different amounts of citric acid and urea were added in order to create a virucidal claim against poliovirus (PV), adenovirus type 5 (AdV) and polyomavirus SV40 (SV40) as non-enveloped test viruses in the presence of fetal calf serum (FCS) as soil load. The exposure time was fixed to 60 s.

**Results:**

With the addition of 2.0 % citric acid and 2.0 % urea an activity against the three test viruses was achieved demonstrating a four log_10_ reduction of viral titers. Furthermore, this formulation was able to inactivate PV, AdV, SV40 and murine norovirus (MNV) in quantitative suspension assays according to German and European Guidelines within 60 s creating a virucidal claim. For inactivation of vaccinia virus and bovine viral diarrhea virus 15 s exposure time were needed to demonstrate a 4 log_10_ reduction resulting in a claim against enveloped viruses. Additionally, it is the first hand disinfectant passing a carrier test with AdV and MNV.

**Conclusions:**

In conclusion, this new formulation with a low alcohol content, citric acid and urea is capable of inactivating all enveloped and non-enveloped viruses as indicated in current guidelines and thereby contributing as valuable addition to the hand disinfection portfolio.

## Background

Virus transfer via human hands is a major route of infection inside and outside medical settings [1]. The efficiency of virus transfer from hands to hands (direct transmission) or via contaminated surfaces (indirect transmission) is closely connected with virus persistence [2]. Detailed studies regarding virus persistence are still lacking however non-enveloped viruses generally are persisting much longer than enveloped-viruses [3–5]. Viruses of the respiratory tract like the non-enveloped rhinovirus and the enveloped viruses like influenza virus and coronavirus can persist on surfaces for several days [6]. When dried, enveloped blood-borne viruses as hepatitis C virus can be infectious for more than a week [7]. Non-enveloped viruses like hepatitis A virus (HAV), adenovirus (AdV) and human norovirus may even persist for several weeks [6, 8]. The actual level of the viral contamination in the environment is likely underestimated due to limited detection of known viruses and presence of unknown viruses [9, 10]. Often the detection of viral genomes by nucleic acid assays is the only way to get insights: Carducci et al. detected viral nucleic acid in 16.7 % of all surface samples in healthcare settings [11]. A disinfectant for hand hygiene therefore would ideally possess broad antiviral spectrum covering non-enveloped in addition to enveloped viruses offering increased protection against persistent, unexpected or unknown viruses [12, 13]. The recommendation of broad spectrum alcohol-based hand rubs (ABHRs) for preventing virus transmission by hands is the most important feature of current guidelines [14].

Worldwide there are differences regarding the number of test viruses needed to be inactivated in standardized tests before a broad spectrum claim including non-enveloped viruses can be given by manufacturers for a hand disinfectant [15]. Regulatory authorities in USA admit activity against certain stable reference viruses using methods of American Society for Testing and Materials (ASTM) (Table 1). The choice of reference test virus is left to the manufacturer [16]. Hand antiseptics can be tested on artificially contaminated hands or fingerpads with test viruses such as AdV, rhinovirus type 14 or 37, human rotavirus, surrogates of human norovirus like MNV or feline calicivirus (FCV) and HAV [17–19]. Yet cases are well documented in which a disinfectant active against a reference virus was not active against an non-enveloped virus like parvovirus [20]. Regulatory authorities in Germany have established a minimum set of test viruses [21] which are not only difficult to inactivate, but also vary in their susceptibility to disinfectants and thus are thought to be representative of the whole known virus families. Under the Guideline of Deutsche Vereinigung zur Bekämpfung der Viruskrankeiten e.V. and the Robert Koch-Institute (DVV/RKI Guideline) [22], disinfectants achieving at least 4 log_10_ titer reduction factor (RF of 4) against vaccinia virus and bovine viral diarrhea virus (BVDV) are active against all enveloped viruses (limited spectrum virucidal) [23, 24]. Disinfectants also inactivating poliovirus (PV), AdV and polyomavirus SV40 (SV40) and since 2015 also MNV can claim activity against all viruses (virucidal) according DVV/RKI Guideline [22]. The discrimination between enveloped only / all viruses was proved successful and has been taken up in the European EN 14476 for hand rubs being valid in whole Europe (Table 1) [25]. In 2012, an additional DVV Guideline for testing the antiviral activity of disinfectants on stainless steel disks carriers simulating practical situations was established [26] and the discrimination limited spectrum virucidal / virucidal claim exists there as well (Table 1). At present, work is in progress to develop a EN Norm for the virucidal carrier test testing MNV and AdV as test viruses [27].

**Table 1:** Overview describing German, European and North Americans norms for virucidal testing

	German Guidelines (DVV/RKI)		European Norms (CEN)		U.S. Methods (ASTM)			
	Suspension test	Carrier test	Suspension test	Carrier test	Suspension test	Carrier test	Fingerpad test	Entire hand test
	DVV/RKI	DVV	EN 14476	prEN 16777	E1052-11	E2197-11	E1838-10	E2011-09
Minimum spectrum of test organisms needed to claim activity against all enveloped viruses (limited spectrum virucidal activity)	BVDV, vaccinia virus	vaccinia virus	murine norovirus, adenovirus	not defined	not defined	not defined	not defined	not defined
Minimum spectrum of test organisms needed to claim activity against all viruses (virucidal activity)	murine norovirus, adenovirus, poliovirus, polyomavirus, SV40	low level: vaccinia virus, adenovirus, murine norovirus, high level: adenovirus, murine norovirus, murine parvovirus	murine norovirus, adenovirus, poliovirus	murine norovirus, adenovirus				
Minimum decimal log reduction needed	4	4	4	4	not defined in the method	not defined in the method	not defined in the method	not defined in the method
Interfering substances for clean conditions	none (Aqua bidest.)	0.3 g/l bovine serum albumin	0.3 g/l bovine serum albumin	0.3 g/l bovine serum albumin	-	-	-	-
Interfering substances for dirty conditions	10 % fetal calf serum (FCS)	3 g/l bovine serum albumin + 3 ml / l sheep erythrocytes	3 g/l bovine serum albumin + 3 ml / l sheep erythrocytes	3 g/l bovine serum albumin + 3 ml / l sheep erythrocytes	5 % bovine serum	5 % bovine serum	5 % bovine serum	5 % bovine serum
Test concentration of rtu product	80 % or 90 %	100 %	80 % or 97 %	100 %		100 %	100 %	100 %

Most biocidal active substances used in hand hygiene have no difficulty inactivating enveloped viruses [1], which are sensitive to alcohol-based hand rubs even in the presence of interfering substances [24]. Achieving the virucidal claim of the DVV/RKI Guideline is considerably more difficult and products that can claim inactivation of all non-enveloped viruses according to the German regulatory model are rare in hand hygiene [28]. The fulfilment of DVV/RKI Guideline and EN 14476 in hand hygiene area is even complicated by the practical requirement of a short exposure time. A user will normally not wait more than about 30–60 s for a ABHRs to act and will not reapply the product if it has dried up before the target exposure time [29].

To date all of the products that claim virucidal activity for hand hygiene under German DVV/RKI Guideline are alcohol-based formulations containing either high amounts of ethanol or an ethanol/1-propanol mixture supplemented with phosphoric acid [30]. Yet high alcohol content hand disinfectants are problematic for reasons of fire safety and toxicity [30] and it is also desirable to produce ABHRs with reduced acidity. Finally, alcohols were reported to inactivate AdV on carriers [31], but little is known regarding the ability of ABHRs to fulfil the DVV carrier test. Therefore, it was the aim to develop a hand disinfectant with a virucidal claim in suspension and carrier tests. We now present a novel ABHR based on ethanol (ca 70 %), supplemented with variable amounts of citric acid and urea additives that fulfils DVV/RKI Guideline and EN 14476 for virucidal activity in quantitative suspension tests. The formulation with the optimum ratio of additives was further characterized in detail showing to possess virucidal activity (without enteroviruses and parvoviruses) on carriers according to DVV Guideline [26].

## Methods

### Viruses and cell cultures

The poliovirus type 1 strain LSc-2ab (Chiron-Behring) was obtained from PD Dr. O. Thraenhart, Eurovir, D-14943 Luckenwalde. The adenovirus type 5 strain Adenoid 75 was obtained from PD Dr. A. Heim, Institute of Medical Virology, Hannover Medical School, D-30625 Hannover. Vaccinia virus strain Elstree (VR-1549, ATCC) originated from the Institute of Medical Virology and Immunology of the University of Essen, D-5122 Essen. Polyomavirus SV40 strain 777 was obtained from PD Dr. A. Sauerbrei, Institute of Virology and Antiviral Chemotherapy at the Friedrich Schiller University of Jena. Murine norovirus S99 (MNV) originated from PD Dr. E. Schreier, Head of FG15 Molecular Epidemiology of Viral Pathogens at the Robert Koch-Institute (RKI) in D-13302 Berlin and BVDV strain NADL (VR-534) was obtained from Dr. S. Bendtfeld, Institute of Virology at the School of Veterinary Medicine Hannover (Tierärztliche Hochschule), D-30559 Hannover.

### Virus propagation

The test virus suspensions were prepared by infecting monolayers of the respective cell lines. The virus titers of these suspensions ranged from 10^7^ to 10^9^ TCID_50_/mL. Poliovirus was propagated in BGM cells (buffalo green monkey = permanent monkey kidney cell line; supplied by Prof. Dr. Lindl, Institut für angewandte Zellkultur, D-81669 München) and adenovirus in A549 cells (human lung epithelial carcinoma cells). The A549 cells originated from the Institute of Medical Virology, Hannover Medical School. Vaccinia virus replication was performed in Vero cells (monkey kidney cell line) obtained from Vircell, SL in ES-18329 Santa Fe, Spain (now BIOTRIN International GmbH, D-69126 Heidelberg). Polyomavirus SV40 was propagated in CV-1 cells (kidney cells of African green monkey) and MVM in A9 cells (mouse cell line, originated from Paul-Ehrlich-Institute, D-63225 Langen). MNV was propagated in RAW 264.7 cells (a macrophage-like, Abelson leukemia virus transformed cell line derived from BALB/c mice, ATCC TIB-71). EKL cells (embryonal cells from bovine lung tissue) for BVDV propagation were used. These cells originated from Mrs. A. Kyas (Henkel KGaA, D-40191 Düsseldorf). Poliovirus and MNV were replicated in Dulbecco’s Modified Eagle’s Medium (DMEM), all other viruses in Eagle’s Minimum Essential Medium with Earle’s BSS (EMEM).

### Biocides

The formulations were supplied by Oro Clean Chemie AG, P.B. 3 32, CH-8320 Fehraltorf, Switzerland containing 69.39 % weight/weight (w/w) ethanol, 3.69 % w/w 2-propanol, different amounts of citric acid ranging from 1.0 to 2.5 % and of urea between 0 % and 2.5 % plus polyethylengylcols as skin care compound. The formulations are manufactured following strict quality criteria. Purified water, prepared by a combination of ion exchange and reverse osmosis from municipal water, was used in preparation of all formulations. The microbial count of purified water was under the 100 CFU/ml acceptance criterion specified in European Pharmacopoeia (Ph. Eur.) 8.0. All other components were of Ph. Eur. quality.

### Quantitative suspension assay

Tests were carried out in accordance with the DVV/RKI Guideline at 20 °C [22]. One part by volume of test virus suspension and one part by volume of Aqua bidest. or FCS were mixed with eight parts by volume of the formulations. Infectivity was stopped by immediate serial dilution with ice-cold medium and later determined by means of end point dilution titration in microtiter plates. 100 μl of each dilution were placed in eight wells of a sterile polystyrene flat bottomed 96-well microtiter plate containing 100 μl suspension of permissive cells. Cultures were observed for cytopathic effects (CPE) after 4–18 days of inoculation depending on the cell culture system. All tests without the initial screening step were conducted in two independent test runs on different days. Virus controls were incorporated after the longest exposure time.

The different formulations of the new hand rub based on ethanol, citric acid and urea were screened undiluted (80.0 % due to the addition of test virus suspension and interfering substance) against PV, AdV and polyomavirus SV40 as non-enveloped test viruses of the Guideline of DVV/RKI in the presence of FCS with a fixed exposure time of 60 s. The ethanol and 2-propanol amounts were constant (69.39 % w/w and 3.69 % w/w, respectively) in these assays while citric acid and urea were used in a dose-dependent manner. The concentration of urea varied between 0 % and 2.5 %, whereas the concentration of citric acid ranged from 1.0 to 2.5 %.

For determination of cytotoxicity the formulations were serially diluted 10-fold in MEM up to a dilution of 10^−5^. One part by volume of water of standardised hardness (instead of test virus suspension) was mixed with one part by volume of interfering substance and eight parts by volume of the disinfectant. Aliquots of 100 μl of each test concentration and each dilution were then inoculated into eight wells of a 96-well microtiter plate containing 100 μl suspension of permissive cells. A control studying the suppression of activity was included. The cell cultures were observed for cytotoxic effects for the same incubation time as afterwards used for the quantitative suspension tests. Virus titers were determined using the methods of Spearman [32] and Kaerber [33] and expressed as log_10_TCID_50_/ml including standard deviation. Titer reduction is presented as the difference between the virus titer after the exposure time with the disinfectant and the control virus titer (water). According to the Guideline of the DVV/RKI, a formulation under test conditions must give at least a 4.0 log_10_ reduction in infectivity titer of test virus (inactivation ≥ 99.99 %) at the recommended concentration and exposure time to be considered active [22, 34].

### Quantitative suspension test according to EN 14476

Tests according to EN 14476 were run in parallel to the Guideline of DVV/RKI with PV, AdV and MNV as test viruses of the EN 14476 and the corresponding permissive cells [25]. The main difference to the German Guideline is the change from Aqua bidest. and FCS as interfering substances to clean conditions (0.3 % bovine serum albumin, final concentration in the test procedure 0.3 g/l) and the use of water for dilutions of ready-to-use products like hand rubs. A control of efficacy for suppression of disinfectant’s activity was included.

### Quantitative carrier test

The quantitative carrier test according to the Guideline of DVV was performed with clean conditions [26]. The cleaning of the stainless steel disks (20 mm diameter, GK Formblech GmbH, D-12277 Berlin, Germany) was performed as described in the Guideline [26]. A total of 50 μl of the virus inoculum was deposited on each pre-treated carrier and dried. Then, inoculum was covered with 100 μl new formulation (for the control 100 μl of hard water was applied) and incubated for 1 and 5 min, respectively. Immediately at the end of the exposure time, the disks were transferred into plastic vial holders (Sarstedt AG & Co. KG, D-51582 Nümbrecht) with 900 μl of ice-cold culture medium to stop the activity of the formulation. Vials were vortexed for 1 min to recover the residual viruses and the eluate was immediately diluted 10-fold (quantal test method) for determining viral infectivity. Cytotoxicity was measured as described in the Guideline [26]. In addition, a control of efficacy for suppression of disinfectant’s activity was included.

### Determination of the slope in a linear regression model for the virucidal effect of urea and citric acid

To estimate differences in the dose-dependency of the virucidal effect of the tested compounds, we calculated the slope of a linear regression model fitted line for urea titration at each citric acid concentration and *vice versa*. Steeper slopes indicate greater dose-dependency.

## Results

### Development and virucidal screening of novel formulations containing constant alcohol and different urea and citric acid concentrations

**Fig. 1:**
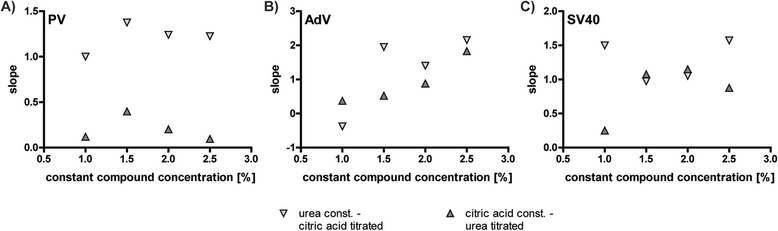
Dose-dependency of virucidal activity of one compound titrated while other compound not was altered. The slope determined via linear regression for urea titration at indicated constant citric acid concentrations (darker triangles) and *vice versa* (lighter triangles) is plotted on the y-axis. The x-axis represents the concentration of the compound held constant. Only values for concentrations ranging from 1.0 % to 2.5 % were taken into account. Higher values point to steeper slopes and thus greater dose-dependency of virucidal effect. Slopes of dose-dependent antiviral effects against PV (**a**), AdV (**b**) and polyomavirus SV40 (SV40) (**c**) are depicted

As shown in Table 2 increasing amounts of citric acid and urea with a constant concentration of ethanol and 2-propanol resulted in a higher virucidal activity (Table 2). For PV the addition of 1.0 % urea and 1.5 % citric acid to the alcohols compounds were sufficient to reach a 4 log_10_ reduction (Table 2). In case of AdV either the combination of 2.0 % urea and 2.0 % citric acid or 1.5 % urea with 2.5 % citric acid were needed to achieve sufficient reduction in viral titers (Table 2). For the polyomavirus SV40 greater virucidal activity as for AdV and a lower activity as for PV with a combination of 1.5 % urea and 1.5 % citric acid could be observed (Table 2). To compare the dose-dependency virucidal effects of urea and citric acid, we calculated the slope of a linear regression model fitted line for urea titration at each citric acid concentration and vice versa. As depicted in Fig. 1 for poliovirus, when the urea concentration was kept constant with increasing citric acid concentrations a clear dose-dependent increase of the virucidal effect could be observed (Fig. 1a). Titration of urea with constant citric acid concentrations did not results in such combinatory effect (Fig. 1a). Similar findings could be observed for AdV and SV40, although here the dose-dependent effect of citric acid was not as pronounced as for PV (Fig. 1b and c). In our system, urea without the addition of acid was not achieving virucidal activity: at 5 % urea the reduction of poliovirus titer was 2.6 log_10_ steps (data not shown). In conclusion, the screening experiments of a novel ethanol-based formulation supplemented with urea and citric acid showed a strong virucidal activity against the three non-enveloped test viruses of the Guideline of DVV/RKI in the presence of FCS.

**Table 2:** Influence of urea and citric acid as additional compounds on virus-inactivating properties of an alcohol-based formulation against poliovirus (PV), adenovirus (AdV) and polyomavirus SV40 (SV40)

PV		concentration of citric acid (%)				
		0.5 %	1.0 %	1.5 %	2.0 %	2.5 %
Concentration of urea (%)	2.5 %	n.d.	3.25 ± 0.58	4.75 ± 0.50	4.88 ± 0.62	5.25 ± 0.51
	2.0 %	n.d.	3.13 ± 0.53	4.13 ± 0.53	4.00 ± 0.54	5.25 ± 0.52
	1.5 %	n.d.	3.13 ± 0.56	4.00 ± 0.58	4.88 ± 0.53	5.13 ± 0.55
	1.0 %	n.d.	n.d.	4.13 ± 0.55	4.25 ± 0.58	5.13 ± 0.52
	0.5 %	n.d.	n.d.	3.00 ± 0.53	3.25 ± 0.50	2.88 ± 0.53
	0 %	n.d.	n.d.	n.d.	3.38 ± 0.55	3.63 ± 0.62
AdV		Concentration of citric acid (%)				
		0.5 %	1.0 %	1.5 %	2.0 %	2.5 %
Concentration of urea (%)	2.5 %	n.d.	2.00 ± 0.66	3.25 ± 0.64	3.88 ± 0.53	5.38 ± 0.65
	2.0 %	n.d.	2.13 ± 0.53	3.00 ± 0.58	4.00 ± 0.46	4.13 ± 0.53
	1.5 %	n.d.	1.63 ± 0.65	2.25 ± 0.57	3.75 ± 0.58	4.38 ± 0.64
	1.0 %	n.d.	n.d.	2.63 ± 0.59	2.50 ± 0.59	2.25 ± 0.68
	0.5 %	n.d.	n.d.	2.63 ± 0.59	2.75 ± 0.56	3.63 ± 0.59
	0 %	n.d.	n.d.	n.d.	3.00 ± 0.65	3.00 ± 0.58
SV40		Concentration of citric acid (%)				
		0.5 %	1.0 %	1.5 %	2.0 %	2.5 %
Concentration of urea (%)	2.5 %	n.d.	3.13 ± 0.52	4.25 ± 0.50	5.00 ± 0.53	5.50 ± 0.27
	2.0 %	n.d.	3.25 ± 0.50	4.88 ± 0.53	4.50 ± 0.38	5.13 ± 0.52
	1.5 %	n.d.	2.88 ± 0.59	4.00 ± 0.59	4.00 ± 0.58	4.50 ± 0.38
	1.0 %	n.d.	n.d.	2.75 ± 0.63	3.25 ± 0.50	4.25 ± 0.50
	0.5 %	n.d.	n.d.	2.88 ± 0.62	3.00 ± 0.58	4.13 ± 0.55
	0 %	n.d.	n.d.	n.d.	3.38 ± 0.45	3.88 ± 0.52

Tests were carried out in a quantitative suspension assay with FCS as interfering substance and 60 s exposure time. Results presented as reduction factor (RF) with 95 % confidence interval. *n.d.* not determined

### Virucidal activity of the final formulation against a broad panel of test viruses

Consequently, the formulation with the sufficient virucidal activity containing 69.39 % w/w ethanol, 3.69 % w/w 2-propanol, 2.0 % urea and 2.0 % citric acid was tested against several non-enveloped (MNV, AdV, PV, polyomavirus SV40) and enveloped viruses (BVDV, vaccinia virus strain Elstree) in the presence or absence of FCS according to Guideline of DVV/RK or in clean conditions according to EN 14476. The results are presented in Table 3 and show that a virucidal activity against enveloped viruses was achieved in already 15 s independent of the soil load. Also the non-enveloped MNV, PV and AdV were inactivated within such a short exposure time of 30 s in clean conditions, whereas 60 s were needed for PV, AdV and polyomavirus SV40 in the presence of FCS (Table 3). These results show that the final formulation supplemented with 2.0 % urea and 2.0 % citric acid exerts a strong virucidal activity against a broad panel of viruses.

**Table 3:** Virucidal activity of the “final formulation” with 2 % urea and 2 % citric acid against test viruses of the German (values with Aqua bidest. and FCS) and European Guidelines (values with clean conditions)

Virus	Conc.	Test method	Soil load	Exposure times			
				15 s	30 s	60 s	90 s
BVDV	80 %	DVV/RKI	Aqua bidest.	≥4.63 ± 0.16	≥4.63 ± 0.16	n.d.	n.d.
BVDV	80 %	DVV/RKI	FCS	≥4.63 ± 0.16	≥4.63 ± 0.16	n.d.	n.d.
Vaccinia virus	80 %	DVV/RKI	Aqua bidest.	≥5.44 ± 0.19	≥5.44 ± 0.19	≥5.44 ± 0.19	n.d.
Vaccinia virus	80 %	DVV/RKI	FCS	≥4.94 ± 0.26	≥5.51 ± 0.18	≥5.51 ± 0.18	n.d.
PV	80 %	DVV/RKI	Aqua bidest.	n.d.	4.32 ± 0.41	≥6.13 ± 0.35	n.d.
PV	80 %	DVV/RKI	FCS	n.d.	3.13 ± 0.37	4.57 ± 0.37	n.d.
PV	80 %	EN 14476	Clean conditions	n.d.	≥5.32 ± 0.34	≥5.75 ± 0.30	n.d.
AdV	80 %	DVV/RKI	Aqua bidest.	n.d.	3.75 ± 0.31	≥4.50 ± 0.29	≥5.13 ± 0.33
AdV	80 %	DVV/RKI	FCS	n.d.	3.44 ± 0.00	4.31 ± 0.42	4.94 ± 0.40
AdV	80 %	EN 14476	Clean conditions	n.d.	4.19 ± 0.31	≥5.38 ± 0.25	n.d.
SV40	80 %	DVV/RKI	Aqua bidest.	n.d.	≥5.44 ± 0.27	≥5.44 ± 0.27	≥5.88 ± 0.29
SV40	80 %	DVV/RKI	FCS	n.d.	3.75 ± 0.55	4.32 ± 0.34	≥5.38 ± 0.25
MNV	80 %	EN 14476	Clean conditions	n.d.	4.13 ± 0.39	≥5.38 ± 0.29	≥5.88 ± 0.33

Results are derived from a quantitative suspension test in duplicates and presented as reduction factor (RF) with 95 % confidence interval. *n.d.* not determined

### Effect of the new formulation with low alcohol content, citric acid and urea against dried viruses

**Fig. 2:**
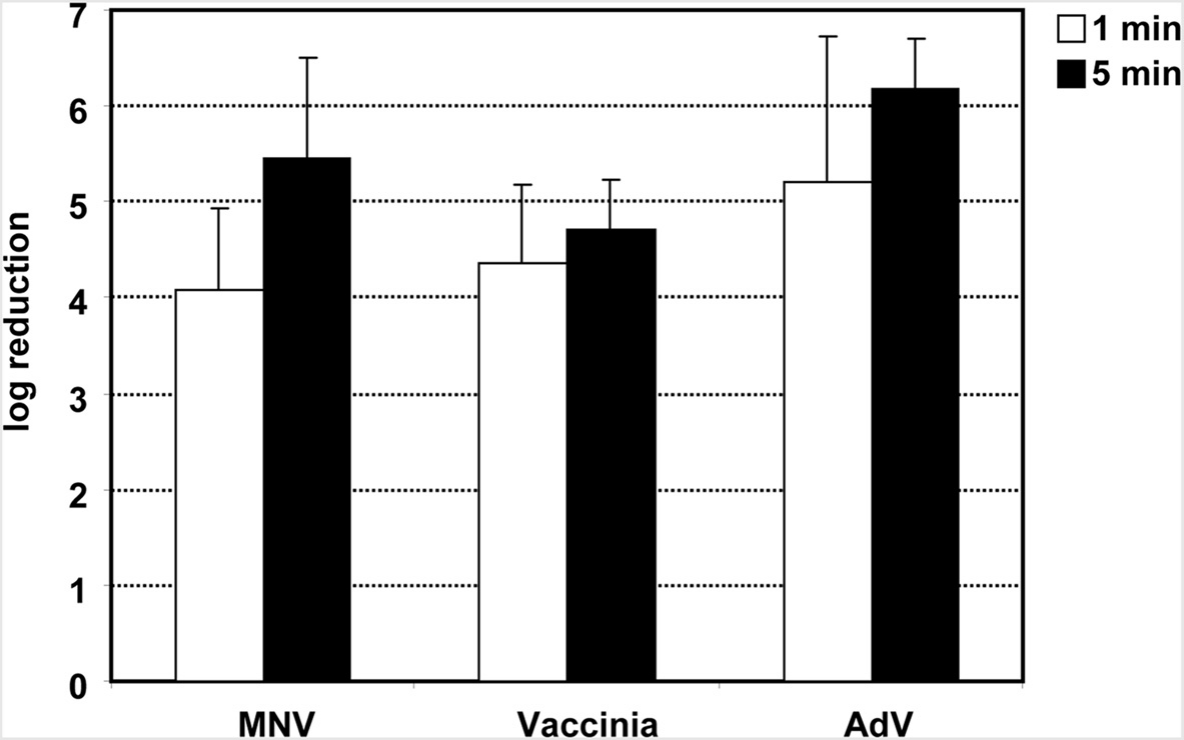
Virucidal efficacy of the final formulation with 2 % urea and 2 % citric acid against dried viruses. A carrier assay was performed with murine norovirus (MNV), vaccinia virus strain Elstree and adenovirus (AdV) as test viruses at two different exposure times. The reduction factor was determined and is displayed with standard deviations

In general, the non-porous surface test method is designed to evaluate the ability of chemical biocides to inactivate vegetative bacteria, viruses, fungi, mycobacteria and bacterial spores on inanimate surfaces. Here, we evaluated the final formulation as described above to inactivate dried vaccinia virus strain Elstree, AdV and MNV as test viruses of the DVV Guideline [35] within 1 and 5 min exposure time on stainless steel disks enabling a virucidal claim (Fig. 2). All test viruses proved to be very stable during the drying process and finally the new formulation achieved the following reduction factors: 4.08 (MNV), 4.37 (vaccinia virus) and 5.21 (AdV) (Fig. 2). Longer exposure times resulted in higher reduction factors for all 3 viruses tested. In conclusion, the alcohol-based formulation containing 69.39 % w/w ethanol, 3.69 % w/w 2-propanol, 2.0 % urea and 2.0 % citric acid proved to be effective not only in a quantitative suspension test, but also against dried viruses thus demonstrating an idea how the product might work at human hands.

## Discussion

The development of broad antiviral spectrum hand disinfectants with activity against all enveloped and non-enveloped viruses is limited by the small number of permissible active ingredients – broad spectrum hand rubs are generally based on alcohol. With ethanol alone in concentrations above 80 % v/v stable non-enveloped viruses like PV and AdV can be inactivated but not polyomavirus SV40 [36, 37]. In contrast, > 60 % v/v 1- and 2-propanol being often used in ABHRs can easily inactivate AdV and SV40 but not PV [36, 38]. Human enterovirus 71 was only inactivated by 95 % ethanol and not by 70 % and 75 % ethanol or any concentration of isopropanol [39]. The reasons for this differential sensitivity of viruses to alcohols are presumed to result from the hydrophobic / hydrophilic nature of the viral particles. The hydrophilic PV is more susceptible to ethanol and the more hydrophobic AdV and polyomavirus SV40 are more susceptible to both propanols [36, 40]. An improvement in activity can be achieved by re-formulation of alcohol solutions with additional ingredients that may enhance the activity. It was therefore a subject of recent developments to look for such additives that would produce a virucidal alcohol-based hand disinfectant. Yet products on the market, which are able to inactivate PV, AdV and MNV as required in the EN 14476 and also polyomavirus SV40 as required by the DVV/RKI Guideline are few. They are based either on 90–94 % ethanol, achieving an activity time of 2 min or about 70 % ethanol with the addition of 0.7 % phosphoric acid, achieving a sufficient activity time of 1 min [30].

We now report that urea in combination with citric acid can enhance the virucidal activity of ethanol solution (ca 70 %) and inactivate all reference viruses of the DVV/RKI Guideline within 1 min exposure time. Tests with bacteria and fungi are still under investigation for this antiseptic formulation which requires a broad spectrum as shown earlier for a product based on chlorine and alcohol [41]. The virucidal activity of concentrated aqueous urea solutions against PV is well known from early experiments with monkeys [42]. Organic acids used as a diluted aqueous solution are active against enveloped but not against non-enveloped viruses on their own [43]. The activity of 70 % ethanol solution against non-enveloped FCV increases from 2.6 log_10_ to >4.4 log_10_ reduction when the pH of the solution is lowered from 7.4 to 3.0 [44]. Citric acid has shown a virucidal efficacy against rhinovirus at artificially contaminated hands [45] and has already been incorporated in an alcohol-based hand rub [46]. Inorganic acids achieve the highest increase in virucidal activity of alcohol formulations [30].

A synergistic virucidal effect of urea and citric acid additives with ethanol was evaluated by measuring the activity against PV, AdV and polyomavirus SV40 according to Guideline of DVV/RKI in the presence of FCS. Keeping urea concentration stable and increasing acid and vice versa, as well as increasing the concentration of both compounds generally increased the antiviral activity of the mixture against all three non-enveloped test viruses. In the presence of FCS, the optimal concentrations of citric acid and urea for the new formulation were identified which resulted in a 4 log_10_ reduction against all three test viruses (Table 2) within 30 to 60 s. Interestingly, we found a better activity in the quantitative suspension test against PV and polyomavirus SV40 compared to AdV with lower concentrations of urea and citric acid. A urea amount of 1.0 % and citric acid amount of 1.5 % was sufficient to inactivate PV and an urea amount of 1.5 % in combination with 1.5 % citric acid was sufficient to inactivate polyomavirus SV, but neither of these formulations was sufficiently active on AdV. These results are consistent with the data from the study of Kramer et al., who tested a virucidal alcoholic hand rub containing a low amount of ethanol and phosphoric acid and found AdV type 2 to be more stable than PV and SV40 [30]. These results strengthen the idea to test viruses from different virus families with various susceptibilities as found in the EN 14476 and DVV/RKI Guideline although it is known that important virus like Hepatitis A Virus and parvoviruses might be more stable than the test viruses used [34].

In Table 2 it can be seen that the formulation containing 2 % citric acid and 2 % urea possesses the needed activity for all required test viruses at the lowest citric acid and urea contents, which was then adopted for further analysis. The virus testing of this formulation confirmed the activity against a broad spectrum of human pathogenic viruses in the quantitative suspension assay. Enveloped viruses like BVDV and vaccinia virus were inactivated within 15 s exposure time. PV, AdV and polyomavirus SV40 were inactivated with A. bidest. and FCS as interfering substances within one minute exposure time. Under clean conditions according to the EN 14476 an exposure time of 30 s was achieved with AdV, PV and MNV. The results also shows that with EN 14476 higher RFs were achieved compared to the DVV/RKI Guideline (Table 3).

Tests on carriers confirmed the activity of the formulation found in the suspension assays also against viruses dried on the surface. The disinfectant inactivated AdV, MNV and vaccinia virus within one minute, making it active against non-enveloped viruses at low level (without enteroviruses and parvoviruses) on carriers, as defined according to DVV Guideline. The activity of the formulation is sufficient for virucidal activity on carriers according to the current version of prEN 16777. Stainless steel carrier methods have shown a good overall reproducibility between different labs [31], but the results on carriers may be not directly transferable to in-vivo situation. Fingerpad methods may be a better alternative for ABHRs testing under practical conditions, however fingerpad methods seem to lack reproducibility, which may be in part due to the inability of the method to properly distinguish the washing out of virus by mechanical means from virus inactivation by disinfectant. When comparing a mixture of propan-1-ol and propan-2-ol (RF = 2.8) and a hand wash product (RF = 3.0) in the fingerpad test, Tuladhar et al. concluded that washing hands with soap and water is better than using hand rubs based on alcohol for removal of norovirus from hands [47]. Own data with the ASTM E1838-10 [17] including modifications derived from the EN 1500 [48] and MNV as test virus demonstrated RFs of 4.25 and 3.94 after 30 s for 2 ethanol-based disinfectants with addition of an acid, whereas even water was able to achieve a RF of 2.86 [49]. Other works report that ABHR in fingerpad tests achieve RFs of 3–4 against non-enveloped viruses, with hard-water rinse achieving RF of 1 [50]. Ethanol-based hand rub fortified with phosphoric acid achieved in the fingerpad test with PV a RF of 3.04 after 30 s [30], whereas 80 % ethanol alone was not active against PV on contaminated hands in earlier tests [51]. Further research should provide clear discrimination between mechanical removal (addition of water) and the additional inactivation by chemical biocides for fingerpad tests. A direct comparison of results from European stainless steel carrier method and artificially contaminated fingerpad or whole hand methods should be carried out.

Hygienic hand disinfection can only be done with intact skin. For dermal tolerance no data for the product developed are available. In another study with a formulation containing 62 % ethanol and 4 % citric acid 9 % of the panelists were not included due to skin irritation [52]. It can be expected that 2 % citric acid will lower the described rate of adverse effect. The amount of urea on human hands after application of the new formulation is unknown. But urea has a positive effect on transepidermal water loss and on skin barrier function [53, 54].

## Conclusion

In conclusion, this new formulation with a low alcohol content, citric acid and urea is capable of inactivating all enveloped and non-enveloped viruses as indicated in EN 14476 and DVV/RKI Guideline in quantitative suspension tests and inactivates MNV, AdV and vaccinia virus on stainless disk carriers. The formulation contributes a valuable addition to the hand disinfection portfolio. It is of course not possible to test the activity of a hand disinfectant against each virus. The test viruses as mentioned in European Norms or German Guidelines analogous to bactericide testing are representatives for the whole spectrum of relevant viruses. Therefore, the new formulation will not only inactivate the test viruses from the European Norm or German Guideline but is also covering the whole spectrum of all enveloped and all non-enveloped viruses being directly or indirectly transferred by human hands.

## Abbreviation

ABHRs: alcohol-based hand rubs
AdV: adenovirus
ASTM: American Society for Testing and Materials
BVDV: bovine viral diarrhea virus
CPE: cytopathic effect
DVV/RKI: Deutsche Vereinigung zur Bekämpfung der Viruskrankheiten e.V. and the Robert Koch -Institute
FCS: fetal calf serum
FCV: feline calicivirus
HAV: hepatitis A virus
MNV: murine norovirus
PV: poliovirus
SV40: polyomavirus SV40
